# Inhibition of Fatty Acid Metabolism Increases EPA and DHA Levels and Protects against Myocardial Ischaemia-Reperfusion Injury in Zucker Rats

**DOI:** 10.1155/2021/7493190

**Published:** 2021-07-28

**Authors:** Janis Kuka, Marina Makrecka-Kuka, Karlis Vilks, Stanislava Korzh, Helena Cirule, Eduards Sevostjanovs, Solveiga Grinberga, Maija Dambrova, Edgars Liepinsh

**Affiliations:** ^1^Latvian Institute of Organic Synthesis, Laboratory of Pharmaceutical Pharmacology, Riga, Latvia; ^2^University of Latvia, Faculty of Biology, Riga, Latvia; ^3^Riga Stradins University, Faculty of Pharmacy, Riga, Latvia

## Abstract

Long-chain *ω*-3 polyunsaturated fatty acids (PUFAs) are known to induce cardiometabolic benefits, but the metabolic pathways of their biosynthesis ensuring sufficient bioavailability require further investigation. Here, we show that a pharmacological decrease in overall fatty acid utilization promotes an increase in the levels of PUFAs and attenuates cardiometabolic disturbances in a Zucker rat metabolic syndrome model. Metabolome analysis showed that inhibition of fatty acid utilization by methyl-GBB increased the concentration of PUFAs but not the total fatty acid levels in plasma. Insulin sensitivity was improved, and the plasma insulin concentration was decreased. Overall, pharmacological modulation of fatty acid handling preserved cardiac glucose and pyruvate oxidation, protected mitochondrial functionality by decreasing long-chain acylcarnitine levels, and decreased myocardial infarct size twofold. Our work shows that partial pharmacological inhibition of fatty acid oxidation is a novel approach to selectively increase the levels of PUFAs and modulate lipid handling to prevent cardiometabolic disturbances.

## 1. Introduction

The combination of interrelated metabolic risk factors such as obesity, dysglycaemia, dyslipidaemia, and insulin resistance creates the basis of metabolic syndrome [1, 2] and promotes the development of cardiovascular diseases and type 2 diabetes mellitus. Metabolic syndrome, when compared to obesity, is related to a higher incidence of major coronary events, including fatal and nonfatal myocardial infarctions [3] and is also associated with increased 20-year mortality in patients [4]. While obesity can be effectively treated by lifestyle and dietary adjustments [5] and/or bariatric surgery [6], a much broader strategy including drug therapy is required to tackle metabolic syndrome [2]. To ensure better translation of novel cardiometabolic therapies to the clinical setting, multiple cardiovascular risk factors and comorbidities should be taken into account when evaluating compounds in a preclinical setting [7–9].

Multiple studies have found an association between the consumption of fish containing omega-3 polyunsaturated fatty acids (PUFAs) and improved cardiovascular outcomes [10]. In contrast, a recent meta-analysis of 23 randomized, double-blind, placebo-controlled trials in cardiovascular patients did not show a protective effect of an additional intake of eicosapentaenoic acid (EPA) and docosahexaenoic acid (DHA) on total mortality and cardiovascular mortality, particularly in the presence of statins [11]. Of interest for the treatment of complications of metabolic syndrome could be finding in a rat model that treatment with *ω*-3 PUFAs decreases the severity of postresuscitation myocardial dysfunction, lipid peroxidation, and systemic inflammation in the early phase of recovery following cardiac arrest [12]. Likewise, transgenic fat-1 mice with constantly elevated levels of endogenous *ω*-3 PUFAs have preserved cardiac contractile function and less inflammation and oxidative stress when challenged with LPS than wild type animals [13]. In insulin-resistant states, an increased reliance of cardiomyocytes on the oxidation of free fatty acids is present and associated with both a lower yield of ATP per oxygen molecule and likely decreased energetic efficiency [14]. It should be noted that complete oxidation of PUFAs is more than 2 times faster than the oxidation of saturated fatty acids [15]. Thus, one can assume that dietary PUFAs are less likely to accumulate to reach the levels/ratios required to induce beneficial effects. In turn, as PUFAs are the preferred substrate [16] for mitochondrial fatty acid metabolism, inhibition of overall fatty acid oxidation could lead to a comparably higher increase in the levels of PUFAs but not saturated fatty acids. Interestingly, plasma lipid parameters are normalized, and the levels of several short- to medium-chain acylcarnitines are significantly decreased by up to 30% after treatment with PUFAs [17]. Altogether, we hypothesized that long-term pharmacological inhibition of carnitine palmitoyltransferase I- (CPT I-) dependent mitochondrial fatty acid metabolism increases the levels of PUFAs and could induce anti-infarction activity in a Zucker rat metabolic syndrome model.

The aim of the present study was to test whether treatment with the carnitine/acylcarnitine-lowering compound methyl-GBB (4-[ethyl(dimethyl)ammonio]butanoate) can decrease overall fatty acid oxidation, thus increasing PUFA availability; and could these alterations in lipid metabolism reduce ischaemia-reperfusion (I/R)-induced cardiac damage in Zucker rats with metabolic syndrome? Zucker rats were used as a representative preclinical model of continuous nutritional overload, physical inactivity, and abdominal obesity, which over time lead to the development of metabolic syndrome [18, 19].

## 2. Materials and Methods

### 2.1. Animals and Treatment

Zucker fa/fa male rats (*n* = 36) and age-matched (5-7-week-old, ~140 g at the time of purchase) Zucker lean male rats (*n* = 18) were obtained from Charles River, and 18 C57BL/6JOlaHsd inbred mice (4-6 weeks old, ~18 g at the time of purchase) were obtained from Envigo. Animals were housed for one week prior to treatment under standard conditions (21-23°C, 12-h light/dark cycle, relative humidity 45-65%) with unlimited access to food R70 diet from Lantmännen and water. The experimental procedures were performed in accordance with the guidelines of the European Community and local laws and policies (Directive 2010/63/EU), and all of the procedures were approved by the Food and Veterinary Service, Riga, Latvia. All experiments were performed in a blinded manner. Studies involving animals are reported in accordance with the ARRIVE guidelines [20, 21].

By limiting the availability of long-chain acylcarnitines (LCAC), methyl-GBB (5–20 mg/kg) significantly decreases infarct size (IS) ex vivo by approximately 50% in normoglycaemic animals [22, 23]. In the present study, a dose of 10 mg/kg was chosen based on previous data regarding methyl-GBB cardiac tissue levels, the effects on carnitine/acylcarnitine levels, cardioprotection in normoglycaemic animals, and data from models with impaired energy metabolism. Although ex vivo methyl-GBB, even at a dose of 5 mg/kg, induces a maximal decrease in IS [23], in previous studies using high-fat diet-fed ApoE^−/−^ knockout mice, methyl-GBB at a dose of 10 mg/kg was required to significantly decrease atherosclerotic lesions and dyslipidaemia [24], two major factors contributing to metabolic syndrome.

Data from previous experiments where I/R damage was determined in methyl-GBB-treated animals were subjected to statistical power analysis, and calculations indicated that *n* = 10 for the infarction study would produce significant results with power > 0.9. Animals were randomly separated into two experimental groups and given daily oral doses of water (Zucker fa/fa control group, *n* = 10) or 10 mg/kg methyl-GBB (Zucker fa/fa methyl-GBB group, *n* = 10) for 12 weeks (18 weeks old at the time of infarction study). Zucker lean rats (*n* = 10) were used as a nonhyperlipidaemic control. To assess mitochondrial levels of acylcarnitines, changes in glucose utilization, molecular pathway upregulation, and mitochondrial functionality, additional experiments were performed in animals treated as described above. Animals were separated randomly into two experimental groups and given daily oral doses of water (Zucker fa/fa control group, *n* = 8) or 10 mg/kg methyl-GBB (Zucker fa/fa methyl-GBB group, *n* = 8). Zucker lean rats (*n* = 8) were used as a nonhyperlipidaemic control. Additional experiments to determine plasma EPA, DHA, and free fatty acid (FFA) concentrations were performed in normoglycaemic C57BL/6 mice. Mice were separated randomly into two experimental groups and given daily oral doses of water (mouse control group, *n* = 9) or 5 mg/kg methyl-GBB (mouse methyl-GBB group, *n* = 9) for 4 weeks. For all experiments, nonfasting or postovernight-fasting blood samples were collected from tail veins; samples were centrifuged, and the plasma was stored at -80°C for future analysis.

### 2.2. Isolated Rat Heart Infarction Study

The infarction was performed according to the Langendorff technique as described previously [25, 26], with some modifications. Animals were anaesthetized with sodium pentobarbital (70 mg/kg), and after the loss of nociceptive reflexes, hearts were immediately excised, thus, sacrificing animals. Hearts were retrogradely perfused with Krebs-Henseleit (KH) buffer solution supplemented with 10 mM glucose (Fresenius Kabi), 0.3 mM sodium palmitate (TCI Europe) bound to 0.5% bovine serum albumin (BSA) (Europa Bioproducts Ltd.), 2 mM lactate (Fischer Scientific), 0.2 mM pyruvate (Acros Organics), and 3 ng/ml insulin aspart (Novo Nordisk) at a constant perfusion pressure of 70 mmHg. After an adaptation period of 20 min, the left anterior descending coronary artery (LAD) was subsequently occluded for 30 min followed by 120 min of reperfusion. IS was determined as described previously [26]. Heart functional parameters, coronary flow, left ventricle developed pressure (LVDP), heart rate, and cardiac workload were registered using the PowerLab system from ADInstruments. Briefly, at the end of reperfusion, the LAD was reoccluded, and the heart was perfused with 0.1% methylene blue dissolved in KH buffer solution to delineate risk and no-risk areas. Afterwards, the ventricles of the heart were transversely cut into 2 mm thick slices and photographed. Computerized planimetric analysis of the stained left-ventricle slice photographs was performed using Image-Pro Plus v6.3 software to determine the area at risk (AR) and the area of necrosis (AN), and each area was expressed as a percentage of the slice area. The obtained values were then used to calculate the IS as a percentage of the risk area, based on the formula IS = AN/*AR* × 100%.

### 2.3. Determination of Glucose Oxidation in Isolated Hearts

The rate of radiolabelled glucose oxidation was measured in additional sets of rat hearts according to a method previously described [22, 23] with the following modifications. Glucose oxidation measurement was performed according to the Langendorff perfusion technique. The hearts were perfused retrogradely (constant perfusion pressure, 70 mmHg) with KH buffer solution supplemented with 10 mM glucose, 0.3 mM sodium palmitate bound to 0.5% bovine serum albumin (BSA), 2 mM lactate, 0.2 mM pyruvate, and 3 ng/ml insulin. After a 10 min adaptation time, the perfusate was switched to the oxygenated radiolabelled KH buffer solution, and perfusion was continued for 10 min at a constant pressure. The glucose oxidation rate was determined by measuring the ^14^CO_2_ released from the metabolism of [U-^14^C]glucose (specific activity, 300 mCi/mmol).

### 2.4. Mitochondrial Energy Metabolism

The mitochondrial energy metabolism was determined in the permeabilized cardiac fibres prepared as described previously [25, 27] with some modifications. The bundles of fibres were permeabilized using 50 *μ*g/ml saponin (Acros Organics) and 0.5 mg/ml collagenase (Sigma-Aldrich) at 4°C in 1 ml of buffer A (20 mM imidazole, 20 mM taurine (all Acros Organics), 0.5 mM dithiothreitol (Tocris Bioscience), 7.1 mM MgCl_2_ (Penta Chemicals), 50 mM MES, 5 mM ATP, 15 mM phosphocreatine, 2.6 mM CaK_2_EGTA, and 7.4 mM K_2_EGTA (all Sigma-Aldrich), pH 7.0 at 0°C). After a 15 min incubation, the fibres were washed for 15 min in 2 ml of buffer B (20 mM imidazole, 0.5 mM dithiothreitol, 20 mM taurine, 1.6 mM MgCl_2_, 100 mM MES, 3 mM KH_2_PO_4_ (Enola Ltd), 2.9 mM CaK_2_EGTA, 7 mM K_2_EGTA, pH 7.1 at 37°C).

High-resolution fluorespirometry was performed using Oxygraph-2 k (O2k; OROBOROS INSTRUMENTS). All experiments were performed at 37°C in MiR05 medium (110 mM sucrose (Enola Ltd.), 60 mM K-lactobionate (Sigma-Aldrich), 0.5 mM EGTA (Sigma-Aldrich), 3 mM MgCl_2_, 20 mM taurine, 10 mM KH_2_PO_4_, 20 mM HEPES (Acros Organics), pH 7.1 at 30°C, and 0.1% essential fatty acid free BSA). The medium was reoxygenated when the oxygen concentration dropped to 80 *μ*M. Palmitoyl-CoA (10 *μ*M) (Larodan AB), carnitine (500 *μ*M for Zucker lean rats and Zucker fa/fa control rats and 20 *μ*M for methyl-GBB-treated rats), and malate (0.5 mM) (Acros Organics) were used to measure CPT I-linked fatty acid oxidation (FAO) dependent on the LEAK state. ADP (Sigma-Aldrich) was added to a concentration of 5 mM to determine oxidative phosphorylation-dependent respiration (OXPHOS state). The FA oxidation-dependent OXPHOS coupling efficiency was calculated as follows:

(1) $$\begin{array}{c} 1–\frac{\text{Resp}.\text{rate LEAK state}}{\text{Resp}.\text{rate OXPHOS state}}. \end{array}$$

Then, pyruvate (5 mM) was added to determine the pyruvate and FA metabolism interaction. The pyruvate-dependent flux control factor was calculated as

(2) $$\begin{array}{c} 1-\frac{\text{Resp}.\text{rate before the addition of pyruvate }\left(\text{PCoA OXPHOS}\right)}{\text{Resp}.\text{rate after the addition of pyruvate }\left(\text{PCoA}+\text{pyruvate OXPHOS}\right)}. \end{array}$$

### 2.5. Determination of Reactive Oxygen Species (ROS) Production

*H_2_O_2_ flux (ROS flux)* in cardiac fibres was measured simultaneously with respirometry using an O2k-Fluorometer and the H_2_O_2_-sensitive probe Ampliflu™ Red (AmR) (Sigma-Aldrich) as described previously [27, 28] with some modifications. First, 10 *μ*M AmR, 1 U/ml horseradish peroxidase (HRP), and 5 U/ml superoxide dismutase (SOD) (all Sigma-Aldrich) were added to the chamber, and H_2_O_2_ detection was performed based on the conversion of AmR to fluorescent resorufin. Calibrations were performed by adding 0.1 *μ*M H_2_O_2_ stepwise. The H_2_O_2_ flux was corrected to take into account the background (AmR slope before sample addition). The respiration protocol was similar to that described above, with the exception that after the assessment of FAO and pyruvate-linked respiration, succinate (10 mM, complex II (CII) substrate) (Sigma-Aldrich) was then added to reconstitute convergent FAO-CI-II-linked respiration. Then, an inhibitor of adenine nucleotide translocator, carboxyatractyloside (Catr) (Sigma-Aldrich), was added to determine the LEAK_Catr_ respiration. Titration with an uncoupler, carbonyl cyanide m-chlorophenyl hydrazine (0.5 *μ*M steps to maximum oxygen consumption) (Sigma-Aldrich), was performed to determine the H_2_O_2_ production rate at the electron transfer system capacity state.

### 2.6. mRNA Isolation and qPCR Analysis

Total RNA from the heart tissues was isolated using TRI reagent (Sigma-Aldrich), and first-strand cDNA synthesis was carried out using a High Capacity cDNA Reverse Transcription Kit (Applied Biosystems™) following the manufacturer's instructions. qPCR analysis of gene expression was performed by mixing SYBR® Green Master Mix (Applied Biosystems™) with synthesized cDNA and forward and reverse primers specific for carnitine palmitoyltransferase IA (CPT IA) and IB (CPT IB), long-chain fatty acid CoA synthetase (ACSL), pyruvate dehydrogenase lipoamide kinase isozyme 4 (PDK4), acyl-CoA oxidase 1 (ACOX), and valosin-containing protein (VCP) and running the reactions on a Bio-Molecular Systems MIC qPCR Cycler according to manufacturer's protocol using following conditions: 95°C for 10 minutes, (95°C for 15 seconds, 60°C for 60 seconds) (60 cycles), 95°C for 60 seconds, followed by melt curve analysis 72-95°C, 0.3°C/sec to ensure amplicon specificity. The relative expression levels for each gene were calculated with the ΔΔ*C*_*t*_ method and normalized to the expression of VCP. The primer sequences used for the qPCR analysis are available upon request.

### 2.7. Western Blot Analysis of Tissue Lysates

Heart muscle tissues were homogenized with an Omni Bead Ruptor 24 homogenizer (Omni International) at a ratio of 1 : 10 (*w*/*v*) at 4°C in a buffer containing 100 mM Tris-HCl (Acros Organics), pH 7.4, 10 mM EDTA (Sigma-Aldrich), 5 mM MgCl_2_, 1 mM glycerol 3-phosphate, 1 mM NaF (all from Fluka), 500 *μ*M Na_3_VO_4_ (Sigma-Aldrich), 1 mM DTT (Tocris Bioscience), phosphatase inhibitor cocktail I 1 : 100 (Alfa Aesar), protease inhibitors (10 *μ*M leupeptin, 1 *μ*M pepstatin (both from Tocris Bioscience), 1 *μ*M aprotinin, and 100 *μ*M AEBSF (both from Sigma-Aldrich)). Total protein was detected using the Lowry method. SDS-PAGE and Western blotting were performed as described previously with some modifications [29]. Briefly, 20 *μ*g of total protein was loaded into Bolt™ 4-12% Bis-Tris Plus Gels (Thermo Scientific). After transfer, the polyvinylidene fluoride membranes (Thermo Scientific) were blocked with 5% BSA (Sigma-Aldrich) in Tris-buffered saline (TBS) for 1 h at room temperature and then incubated overnight at 4°C with primary antibodies. After washing with TBS, the blots were incubated for 1 h at room temperature with secondary peroxidase-coupled goat anti-rabbit IgG (lot: 26, #7074 Cell Signaling Technology) and then washed again with TBS. The blots were developed using chemiluminescence reagents (Millipore) and an Azure c400 Imaging System. The Western blot images were analysed using AzureSpot2.0 software. The phosphorylation levels of Akt at Ser473 on the membranes were detected with a phospho-Akt Ser473 (lot: 23, #4060 Cell Signaling Technology) antibody, and the obtained data were normalized to the total Akt level (lot: 20, #4691 Cell Signaling Technology).

### 2.8. Determination of Biochemical Measures

The plasma insulin concentrations were determined using a Sensitive Rat Insulin RIA Kit. Blood glucose was measured using a MediSense Optium glucometer from Abbott Diabetes Care. The concentration of FFAs was determined using a kit from Wako in plasma, and lactate was determined using a kit from Roche Diagnostics. Triglycerides, total cholesterol, aspartate aminotransferase (ASAT), and alanine aminotransferase (ALAT) in plasma were determined using kits from Instrumentation Laboratory. HOMA-IR was calculated according to the formula

(3) $$\begin{array}{c} \frac{\text{fasting insulin }\left(\mu \text{U}/\text{L}\right)\text{ x fasting glucose}}{22.5}. \end{array}$$

### 2.9. Measurement of Carnitine, Methyl-GBB, Acylcarnitines, and Poly-Unsaturated Fatty Acids by UPLC/MS/MS and Metabolomics

The determination of carnitine and methyl-GBB in heart tissues and plasma samples was performed by ultraperformance liquid chromatography-tandem mass spectrometry (UPLC/MS/MS) using the positive ion electrospray mode [23]. Mitochondria samples were prepared as previously described [30], and acylcarnitines were analysed by the UPLC MS/MS method [29]. PUFAs were measured as follows: 200 *μ*l of acetonitrile–methanol mixture (3 : 1, *v*/*v*) were added to 40 *μ*l of plasma samples or calibration sample solution. The samples were thoroughly mixed and centrifuged for 10 min at 10000 rpm, and the supernatants were subjected to UPLC/MS/MS analysis. The analysis was performed on Acquity UPLC system coupled with Xevo TQ-S microtriple-quadrupole mass spectrometer (Waters Corp.). The separation of analytes was carried out on an Acquity UPLC BEH C18 column (2.1 × 50 mm, 1.7 *μ*m, Waters Corp.) operated at 30°C under a gradient elution from 40 to 98% acetonitrile in 0.1% FA. Flow rate was 0.4 ml/min, and injection volume was 1 *μ*l. The mass spectrometer was operated using an electrospray ionization source in negative ion mode. The multiple reaction monitoring (MRM) mode was employed for quantitative determination of target compounds. The main ionization settings were optimized as follows: capillary voltage of 2.0 kV; source and desolvation temperatures of 140 and 600°C, respectively; desolvation gas (nitrogen) flow of 800 L/h. Cone voltage (V) and collision energy (eV) values were specified for each compound: 20 and 15 for EPA; 20 and 12 for DHA, respectively. Quantitative analysis was performed under MRM mode by calculating the peak areas. Precursor to product ion transitions *m*/*z* 301.29 → 203.31 and 301.29 → 257.45 for EPA and *m*/*z* 327.30 → 229.35 and 327.30 → 283.35 for DHA were monitored. Data acquisition and processing were performed using the MassLynx V4.1 and QuanLynx V4.1 software (Waters Corp.). The samples were kept at 10°C in the autosampler. The calibrators were run in triplicate and the samples in duplicate. Concentrations of EPA, DHA, and FFAs (C12-C20) were measured in mice plasma samples by Biocrates Life Sciences AG (Innsbruck, Austria) as a part of their metabolomics analysis service by using Biocrates MxP Quant 500 Kit.

### 2.10. Statistical Analysis

The data are presented as the mean ± S.E.M. (standard error of the mean). Based on a normality test (D'Agostino & Pearson omnibus normality test (Shapiro-Wilk normality test if *N* = 7 or lower)) of the data, statistically significant differences in the mean values were evaluated using one-way ANOVA or the Kruskal-Wallis test. If ANOVA indicated *P* < 0.05, Tukey's test was performed, and the differences were considered significant when *P* < 0.05. If the Kruskal-Wallis test indicated *P* < 0.05, Dunn's multiple comparison test was performed, and the differences were considered significant when *P* < 0.05. The data were analysed using GraphPad Prism statistical software (GraphPad Inc., USA). For IS determination, 1 heart could not be analysed in the methyl-GBB-treated rat group, and 3 hearts could not be analysed in the Zucker lean rat group due to isolated heart perfusion system tubing failure. To determine functional heart parameters, LCAC levels, and metabolic patterns, an additional experiment was performed; 1 sample could not be analysed in the Zucker fa/fa rat control group, as the animal died suddenly of natural causes, and the data represent the mean values of *N* = 7 for this group. The data and statistical analyses comply with the recommendations for experimental design and analysis in pharmacology [31].

### 2.11. Materials

Methyl-GBB (a phosphate salt) was obtained from JSC Grindeks (Riga, Latvia). An insulin RIA kit was purchased from Millipore (USA) and [U-^14^C]glucose was purchased from Biotrend Chemikalien GmbH (Germany).

## 3. Results

### 3.1. Plasma Concentrations of EPA, DHA, and Lipid Species

**Figure 1:**
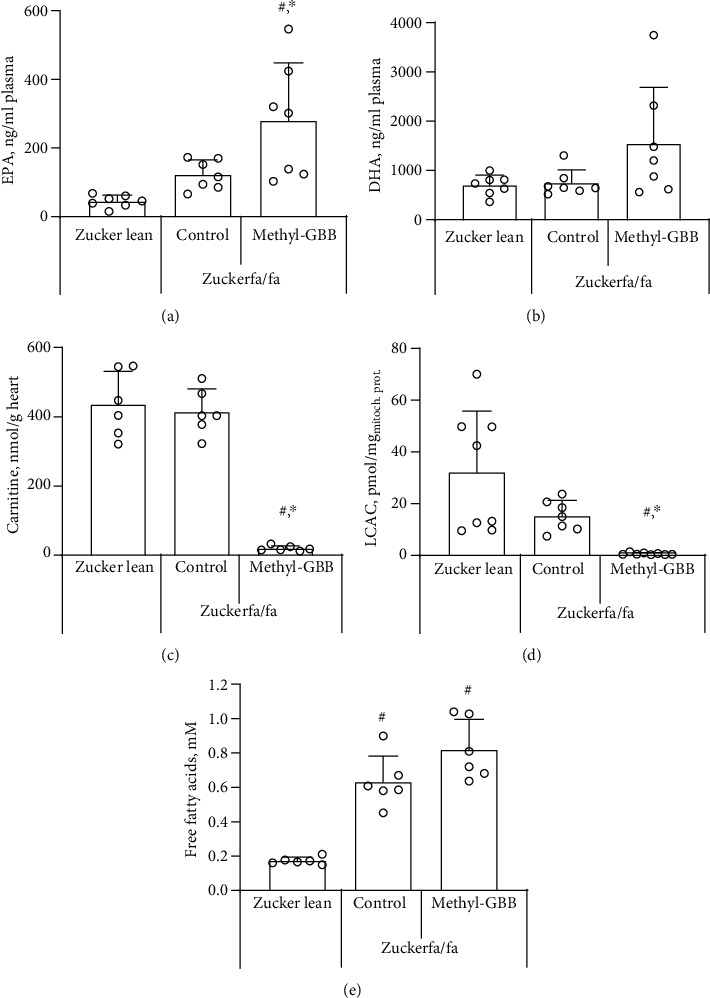
The effect of methyl-GBB administration on plasma EPA (a) and DHA (b) concentrations, cardiac tissue carnitine content (c), mitochondrial LCAC content (d), and plasma free fatty acid concentration (e) after 12 weeks of treatment. Each value was calculated as the mean ± S.E.M. of 7 rats for (a) and (b), 6 rats for (c) and (e), and 8(7) rats for (d). ^∗^Significantly different from the Zucker fa/fa control group; ^#^significantly different from the Zucker lean rat group (ANOVA followed by Tukey's test; *P* < 0.05).

First, data on EPA and DHA plasma concentrations were obtained from normoglycaemic mice treated with methyl-GBB for 4 weeks at a dose of 5 mg/kg. EPA and DHA plasma concentrations in methyl-GBB-treated mice were significantly increased 3.4- and 1.9-fold in plasma from fasted mice, respectively, and 2.5- and 2.3-fold in plasma from fed mice, respectively (Supplementary Figure 1). Moreover, the concentration of FFAs (sum of C12-C20) was 1.3-fold increased only in fasted mouse plasma after treatment with methyl-GBB (Supplementary Figure 2). Next, in Zucker rats, plasma concentrations of EPA and DHA were measured after 12 weeks of treatment with methyl-GBB at a dose of 10 mg/kg. No significant difference was observed between EPA levels in the Zucker lean and Zucker fa/fa rat control groups, although there was a tendency for higher EPA concentrations in the Zucker fa/fa control group. Methyl-GBB treatment resulted in a significant 6.3- and 2.3-fold increase in the plasma EPA concentration compared to the Zucker lean rat and Zucker fa/fa rat control groups (Figure 1(a)). DHA levels were similar in the Zucker lean and Zucker fa/fa control groups. Treatment with methyl-GBB increased the DHA concentration 2.1-fold, but the effect was not statistically significant (Figure 1(b)). The carnitine levels in the Zucker lean and Zucker fa/fa rat hearts were similar, while in the methyl-GBB-treated rat hearts, the carnitine level was significantly decreased by 95% (Figure 1(c)). The LCAC content was determined in isolated cardiac mitochondria. Interestingly, the LCAC content in isolated cardiac mitochondria after I/R was 52% lower in the Zucker fa/fa control rat group than in the Zucker lean rat group; however, this effect was not statistically significant. Treatment with methyl-GBB significantly decreased the heart mitochondrial content of LCAC 25-fold compared to the Zucker fa/fa control group (Figure 1(d)).

In the fed state, the concentration of FFAs in plasma was significantly higher in the Zucker fa/fa control and methyl-GBB-treated Zucker fa/fa rats than in the Zucker lean rats (Figure 1(e)). Similarly, the concentrations of triglycerides and total cholesterol in plasma were significantly higher in the fed Zucker fa/fa control and methyl-GBB-treated Zucker fa/fa rats than in the Zucker lean rats (Table 1). Methyl-GBB treatment had no significant effect on the ASAT and ALAT levels when compared to the Zucker fa/fa rat control group. While methyl-GBB treatment significantly increased the PUFA content by up to 2.3-fold (Figures 1(a) and 1(b)), only a nonsignificant tendency to increase the total FFA concentration by 30% in the methyl-GBB-treated group was observed when compared to the Zucker fa/fa control group (Figure 1(e)). Zucker fa/fa rats gained significantly more weight than Zucker lean rats, and methyl-GBB had no effect on weight gain or on the organ-to-body weight ratio. Overall, while treatment with methyl-GBB significantly increased plasma concentrations of PUFAs, it had no effect on other lipid species.

### 3.2. Isolated Rat Heart Infarction and Haemodynamic Parameters and Mitochondrial Function after Ischaemic Damage

**Figure 2:**
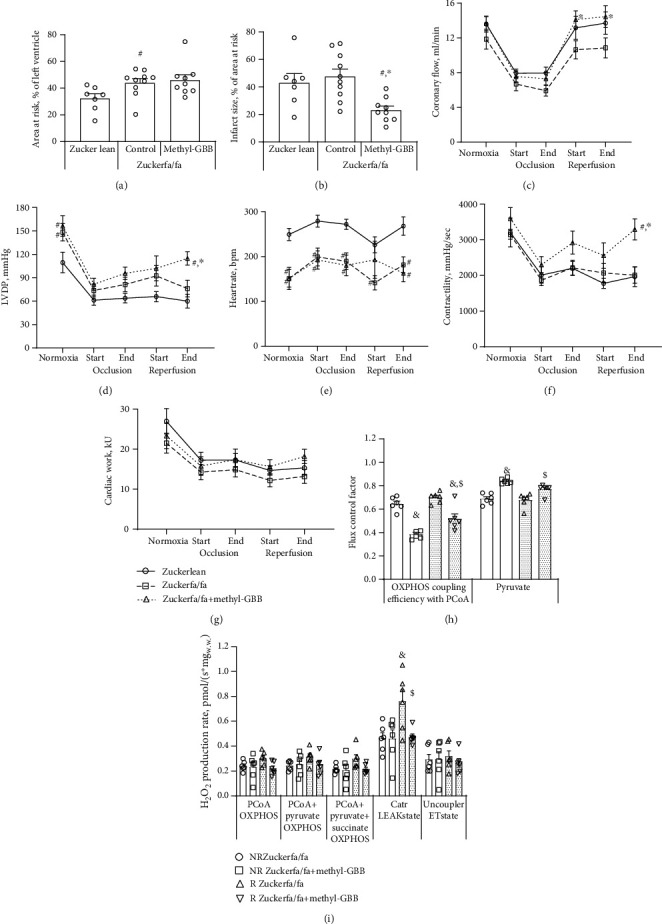
The effect of methyl-GBB administration on myocardial infarct size (a, b) and heart functional parameters (c–g) in Langendorff-perfused isolated hearts and the flux control factor (h) and mitochondrial ROS production (i) in permeabilized cardiac fibres from normoxic nonrisk (NR) and reperfused area at risk (R) regions of the heart after 12 weeks of treatment. Each value was calculated as the mean ± S.E.M. of 7 hearts in the Zucker lean group, 9 hearts in the methyl-GBB-treated group, and 10 hearts in the Zucker fa/fa control group for (a) and (b) and 8(7) hearts for (c–g), and 6 hearts for (h) and (i). ^∗^Significantly different from the Zucker fa/fa control group; ^#^significantly different from the Zucker lean rat group (Kruskal-Wallis test followed by Dunn's multiple comparison test; *P* < 0.05 for (a), ANOVA followed by Tukey's test; *P* < 0.05 for (b), two-way ANOVA followed by Tukey's test; *P* < 0.05 for (c–g)). ^$^Significantly different from the respective Zucker fa/fa control (NR or R) group. ^&^Significantly different from the respective normoxic (NR) group (ANOVA followed by Tukey's test; *P* < 0.05).

A heart infarction study was performed after 12 weeks of treatment with methyl-GBB at a dose of 10 mg/kg. Treatment with methyl-GBB significantly decreased the myocardial IS by 51% (Figure 2(b)) compared to the Zucker fa/fa rat control group. The IS in the methyl-GBB-treated group was also significantly lower (46%) than that of the Zucker lean rat group. There was no statistically significant difference between the area at risk (AR) values in the Zucker fa/fa rat control group and in the methyl-GBB-treated Zucker fa/fa groups. The AR value in the Zucker fa/fa control group was significantly larger by 36% than that in the Zucker lean rat group (Figure 2(a)). As in previous studies in healthy animals, methyl-GBB treatment had no significant effect on normoxic heart functional parameters in Zucker fa/fa rats when compared to the Zucker fa/fa control group (Figures 2(c)–2(g)). Coronary flow during LAD occlusion was decreased by 40% in the Zucker lean rat group and by 43% and 45% in the Zucker fa/fa control and methyl-GBB-treated groups, respectively (Figure 2(c)). During reperfusion, recovery of coronary flow was significantly better in the methyl-GBB-treated group than in the Zucker fa/fa control group. During reperfusion, LVDP (Figure 2(d)) was significantly higher in methyl-GBB-treated rat hearts than in Zucker lean and Zucker fa/fa rat hearts, and a tendency to preserve cardiac workload (Figure 2(g)) was observed in methyl-GBB-treated Zucker rat hearts. Methyl-GBB treatment had no effect on heart rate when compared to the Zucker fa/fa control group (Figure 2(e)). Heart contractility at the end of reperfusion was significantly higher by 67 and 64% in the methyl-GBB-treated group than in the Zucker lean rat and Zucker fa/fa control groups, respectively (Figure 2(f)).

To evaluate the effects of methyl-GBB treatment on mitochondrial functionality after ischaemic damage, permeabilized cardiac fibres were compared from both nonrisk (NR) and AR regions of the heart. In the presence of carnitine concentrations that mimic the tissue carnitine content, methyl-GBB decreased the CPT I-dependent OXPHOS coupling efficiency with palmitoyl-coenzyme A (PCoA) in normoxic cardiac fibres and after reperfusion, indicating an overall decrease in FAO (Figure 2(h)). Interestingly, in the Zucker fa/fa control group after reperfusion, no increase in the PCoA-dependent OXPHOS coupling efficiency was observed when risk and normoxic nonrisk areas were compared. In contrast, fatty acid metabolism during reperfusion was increased by 34% in cardiac fibres from AR regions from methyl-GBB-treated rat hearts compared to normoxic NR areas from these hearts. In addition, methyl-GBB significantly increased pyruvate metabolism in both normoxic and reperfused tissues (Figure 2(h)). I/R tended to increase mitochondrial reactive oxygen species (ROS) production in the OXPHOS state and significantly increased ROS production in the LEAK_Catr_ state compared to normoxic conditions. Methyl-GBB treatment prevented an increase in ROS production, particularly in the LEAK_Catr_ state (Figure 2(i)). Together, treatment with methyl-GBB optimizes energy metabolism and preserves mitochondrial functionality.

### 3.3. Insulin Sensitivity and Energy Metabolism Regulation

**Figure 3:**
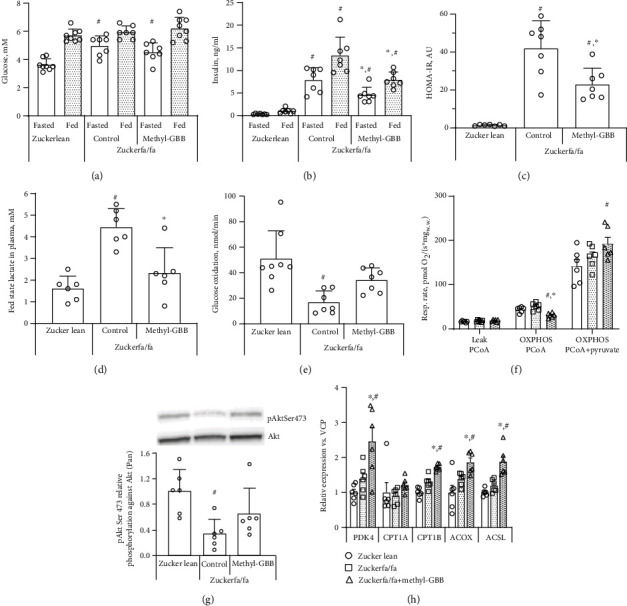
The effects of methyl-GBB administration on blood glucose (a), insulin (b), HOMA-IR (c), plasma lactate (d) concentrations, glucose oxidation in isolated Langendorff-perfused rat hearts (e), and pyruvate and CPT I-dependent fatty acid metabolism in cardiac fibres (f) under normoxic conditions, Akt phosphorylation (g), and the expression of genes related to fatty acid and glucose metabolism (h) after 12 weeks of treatment. Representative Western blots for Akt phosphorylation are presented in (g). Each value was calculated as the mean ± S.E.M. of 6 rats for (d) and (f–h) and of 7-8 rats for (a–c) and (e). ^∗^Significantly different from the Zucker fa/fa control group (ANOVA followed by Tukey's test/Kruskal-Wallis followed by Dunn's test; *P* < 0.05). ^#^Significantly different from the Zucker lean group (ANOVA followed by Tukey's test/Kruskal-Wallis followed by Dunn's test; *P* < 0.05).

The glucose concentration was similar in fed Zucker lean and Zucker fa/fa rat blood (Figure 3(a)). In the fasted state, the blood glucose concentration in Zucker fa/fa rats was significantly higher (26%) than that of fasted Zucker lean rats (Figure 3(a)). In addition, insulin concentrations were significantly 22- and 13-fold higher in Zucker fa/fa rat plasma than in Zucker lean rats in the fasted and fed states, respectively. Treatment with methyl-GBB significantly decreased plasma insulin concentrations by 42% and 40% in the fasted and fed states, respectively (Figure 3(b)). The homeostasis model assessment of insulin resistance (HOMA-IR) value for healthy Zucker lean rats was 1.38, while it was 30.5-fold higher in the Zucker fa/fa control group. Treatment with methyl-GBB significantly decreased the HOMA-IR value in Zucker fa/fa rats by 1.8-fold compared to the Zucker fa/fa control group (Figure 3(c)). The lactate plasma concentration was determined in the fed state and was significantly higher (2.5-fold) in the Zucker fa/fa rat control group than in the Zucker lean rats; however, treatment with methyl-GBB significantly decreased the plasma lactate level by 48%, indicating more complete glucose metabolism (Figure 3(d)).

Because methyl-GBB treatment was found to improve insulin sensitivity in Zucker fa/fa rats, the glucose oxidation rate was measured in isolated perfused normoxic rat hearts. Glucose utilization was significantly impaired in Zucker fa/fa control rat hearts, with a 2.7-fold decrease compared to that in Zucker lean rat hearts. Treatment with methyl-GBB increased glucose utilization close to the level observed in the Zucker lean rat group (Figure 3(e)). Mitochondrial fatty acid and pyruvate metabolism were evaluated in cardiac fibres from nonischaemic areas of the heart. Methyl-GBB treatment significantly decreased the CPT I-dependent oxidative phosphorylation- (OXPHOS-) coupled mitochondrial metabolism of long-chain PCoA by 40% compared to that of the Zucker fa/fa control group (Figure 3(f)). In addition, OXPHOS-coupled pyruvate metabolism was significantly facilitated in methyl-GBB-treated Zucker rats compared to Zucker lean rats (Figure 3(f)).

We measured the protein phosphorylation levels of phosphorylated Akt (P-Akt) and standardized them to the total Akt level. The P-Akt level was significantly lower in the Zucker fa/fa control group than in the Zucker lean group. The P-Akt level in methyl-GBB-treated rat hearts was close to that in Zucker lean rat hearts (Figure 3(g)). The effects of methyl-GBB on the expression of PPAR*α*/PGC1*α* target genes related to fatty acid and glucose metabolism were determined in cardiac tissues. Treatment with methyl-GBB significantly increased the expression of CPT IB, ACOX, and ACSL up to 1.9-fold when compared to both Zucker lean and Zucker fa/fa control rats (Figure 3(h)). Moreover, the expression of the PDK4 gene was significantly upregulated 2.5-fold in methyl-GBB-treated rat hearts, indicating activation of the PPAR*α*/PGC1*α* pathway (Figure 3(h)). Together, treatment with methyl-GBB improves insulin sensing, decreases fatty acid levels, and, in turn, increases glucose metabolism.

## 4. Discussion

The present study shows that the cardioprotective drug methyl-GBB can decrease I/R damage, diminish energy metabolism dysfunction in mitochondria, and reduce glucose-lactate metabolism disturbances in Zucker rats with metabolic syndrome. The data for the first time show that a significant decrease in fatty acid oxidation in mitochondria by methyl-GBB treatment results in increased plasma availability of PUFAs. Moreover, an increase in PUFA availability was also observed in normoglycaemic mice treated with methyl-GBB, indicating that a pharmacological decrease in fatty acid metabolism is a viable option to further increase PUFA availability.

Treatment with methyl-GBB increases plasma concentrations of PUFAs, particularly that of EPA, and decreases the levels of acylcarnitines. Previous studies have shown that long-term treatment with PUFAs provides cardioprotection in rats in vivo [32]. While CD36-mediated transport is pivotal for FA oxidation to support myocardial contractile function [33], lipotoxicity, in turn, is a well-known paradigm based on cardiac imbalance between reduced utilization of FAs and noninterrupted FA delivery [34]. Thus, ongoing CPT I-dependent metabolism of FAs and subsequent accumulation of saturated LCAC is known to impair mitochondrial functionality, which further damages the myocardium after I/R [35, 36]. In a recent study, we demonstrated that the accumulation of LCAC in mitochondria during ischaemia inhibits ATP synthesis, enhances ROS production, and induces I/R damage [30]. Here, we show that pharmacologically limited fatty acid oxidation in methyl-GBB-treated Zucker fa/fa rats decreases the LCAC content in cardiac mitochondria and elevates PUFA levels. These changes are accompanied by the prevention of I/R-induced increases in ROS production. As a result, a marked 51% decrease in IS is achieved even in obese Zucker rats with metabolic syndrome and severely impaired energy metabolism.

LCAC accumulation induces insulin resistance by inhibiting the Akt-mediated signalling pathway [29]. Recently, we showed that methyl-GBB, by decreasing LCAC tissue levels, improves insulin sensitivity and glucose tolerance test responses [37]. Moreover, the levels of PUFAs have a strong positive correlation with insulin sensitivity [38], and improvement in insulin sensitivity after treatment with EPA was shown to result in functional improvements in glucose and fatty acid uptake and cardiomyocyte shortening [39]. In the present study, Akt phosphorylation was preserved, insulin sensitivity was significantly improved, and glucose and pyruvate metabolism was facilitated. Akt phosphorylation is another parameter that could be increased not only due to the decrease in LCAC availability but also due to the increase in PUFA availability [40]. Overall, pharmacological limitation of fatty acid oxidation improves insulin sensitivity in Zucker rats.

In insulin-resistant tissues, the accumulation of lipids and their metabolites inhibits the metabolism of pyruvate [41, 42]; this leads to a situation where glycolysis is not coupled to oxidative phosphorylation. As a result, the lactate concentration in plasma is increased to the level typical of the lactate threshold induced by excessive physical activities. In this state, pyruvate oxidation in the mitochondria is significantly decreased, followed by the formation of lactate and elevated lactate plasma concentrations [37, 43–45]. In morbidly obese Zucker rats, the lactate plasma concentration is increased almost 3-fold, while methyl-GBB treatment significantly improves pyruvate metabolism in mitochondria and decreases the lactate concentration almost to the control level. Recently, we confirmed that the effects related to insulin sensitization and the facilitation of pyruvate and lactate oxidation depend mostly on a decrease in the LCAC content in muscles [29, 41], while the current study for the first time confirms that these effects also apply to cardiac tissues from animals with metabolic syndrome. Overall, facilitation of glucose metabolism by the activation of pyruvate metabolism in mitochondria could also be partially responsible for the anti-infarction effect of methyl-GBB.

Current findings further indicate that treatment with methyl-GBB prevents an I/R-induced increase in mitochondrial ROS production under conditions of limited oxidative phosphorylation. Since it has been shown that the accumulation of LCAC in ischaemic cardiac mitochondria promotes ROS production due to the inhibition of oxidative phosphorylation [30, 46], the observed prevention of oxidative stress in ischaemic hearts by methyl-GBB treatment is more likely associated with the prevention of LCAC accumulation during ischaemia. Moreover, activation of mitochondrial respiration and FA oxidation decreases lipotoxicity [47], and it has been suggested that stimulation of fatty acid oxidation during reperfusion could be beneficial against I/R-induced oxidative stress [48]. Treatment with methyl-GBB increases mitochondrial fatty acid oxidation after ischaemia, thus ensuring effective utilization of accumulated fatty acid intermediates and subsequent reduction of oxidative stress. Overall, these results indicate that a pharmacological decrease in the LCAC content in cardiac mitochondria attenuates ROS production under conditions of limited oxidative phosphorylation after I/R and protects against myocardial damage.

Previously, it was shown that methyl-GBB limits I/R damage in animals with intact glucose metabolism [22, 23], while the present findings extend the cardioprotective effect to conditions with markedly impaired cardiac glucose metabolism, such as metabolic syndrome. The current results provide additional proof that the cardioprotective effect of methyl-GBB is related to changes in fatty acid handling. Thus, EPA and DHA each can protect the heart against myocardial infarction damage [40], and treatment with methyl-GBB increases the availability of the aforementioned PUFAs. In addition, earlier observations by Harmancey and colleagues [49] indicate that the improved utilization of long-chain fatty acids during reperfusion leads to better recovery in insulin-resistant hearts. While treatment with methyl-GBB decreases CPT I-dependent long-chain fatty acid conversion to LCAC and overall fatty acid metabolism, a significant increase in fatty acid mitochondrial utilization in areas at risk compared to that in normoxic areas from the same heart was observed in methyl-GBB-treated hearts after reperfusion but not in control Zucker fa/fa rat hearts. Changes in the fatty acid metabolism pattern are accompanied by improved cardiac functionality in methyl-GBB-treated Langendorff-perfused isolated Zucker fa/fa rat hearts during reperfusion. This effect was not observed before in normoglycaemic animals [22, 23]. Significant increases in the expression of several PPAR*α*/PGC1*α* target genes involved in fatty acid oxidation might at least partially be the result of increased PUFA levels [50, 51] and could be responsible for increased fatty acid oxidation recovery in reperfused tissues.

## 5. Conclusions

The current study highlights the potential benefits of pharmacologically increasing PUFAs and decreasing LCAC levels by methyl-GBB to improve insulin sensitivity and mitochondrial functionality as well as the cardioprotective effect of methyl-GBB in treating metabolic syndrome. Presented here is a strategy to accumulate PUFAs and to facilitate overall fatty acid metabolism during reperfusion to decrease LCAC availability. This leads to the protection of the myocardium against damage induced by ischaemia under complex metabolic conditions and raises expectations for the successful translation of such a strategy to clinical settings.

## Figures and Tables

**Table 1:** The effects of methyl-GBB administration on triglyceride, total cholesterol, ASAT and ALAT concentrations in fed rat plasma, and weight gain after 12 weeks of treatment.

	Zucker lean	Zucker fa/fa	
		Control	Methyl-GBB 10 mg/kg
Triglycerides, mM	0.18 ± 0.02	1.55 ± 0.23^#^	1.56 ± 0.52^#^
Total cholesterol, mg/100 ml	48 ± 4	108 ± 3^#^	113 ± 7^#^
ASAT, U/l	47.6 ± 1.5	48.7 ± 3.5	45.9 ± 1.3
ALAT, U/l	23.5 ± 1.7	39.8 ± 3.0^#^	38.7 ± 2.1^#^
Body weight gain, g	+241 ± 10	+414 ± 10^#^	+390 ± 14^#^
Heart to body weight, mg/g	3.70 ± 0.23	2.97 ± 0.09^#^	2.97 ± 0.10^#^
Liver to body weight, mg/g	34.4 ± 0.6	36.4 ± 1.1	35.4 ± 0.7
Kidney to body weight, mg/g	5.82 ± 0.07	4.61 ± 0.08	4.47 ± 0.08

Each value was calculated as the mean ± S.E.M. of 6 rats for triglyceride and total cholesterol concentrations, 8 rats for ASAT and ALAT levels, and 10 rats for body weight gain. ^#^Significantly different from the Zucker lean group (ANOVA followed by Tukey's test; *P* < 0.05).

## Data Availability

The datasets analysed during the current study are available from the corresponding author on reasonable request.

## Supplementary Materials

Supplementary Materials Supplementary Figure 1: the effect of methyl-GBB administration on mouse plasma EPA (A) and DHA (B) concentrations after 4 weeks of treatment at a dose of 5 mg/kg. Each value was calculated as the mean ± S.E.M. of 9 mice. ^∗^Significantly different from the respective fed or fasted control group (ANOVA followed by Tukey's test; *P* < 0.05). Supplementary Figure 2: the effect of methyl-GBB administration on mouse plasma FFA concentration (sum of C12-C20 FAs) after 4 weeks of treatment at a dose of 5 mg/kg. Each value was calculated as the mean ± S.E.M. of 9 mice. ^∗^Significantly different from the respective fasted control group; # (ANOVA followed by Tukey's test; *P* < 0.05).
