# Supplementary material for: Inhibition of Fatty Acid Metabolism Increases EPA and DHA Levels and Protects against Myocardial Ischaemia-Reperfusion Injury in Zucker Rats
Source: Oxid Med Cell Longev. 2021 Jul 28;2021:7493190. doi: 10.1155/2021/7493190 (PMC8342141; doi:10.1155/2021/7493190)
Supplement: Supplementary Materials — Supplementary Figure 1: the effect of methyl-GBB administration on mouse plasma EPA (A) and DHA (B) concentrations after 4 weeks of treatment at a dose of 5 mg/kg. Each value was calculated as the mean ± S.E.M. of 9 mice. ∗Significantly different from the respective fed or fasted control group (ANOVA followed by Tukey's test; P < 0.05). Supplementary Figure 2: the effect of methyl-GBB administration on mouse plasma FFA concentration (sum of C12-C20 FAs) after 4 weeks of treatment at a dose of 5 mg/kg. Each value was calculated as the mean ± S.E.M. of 9 mice. ∗Significantly different from the respective fasted control group; # (ANOVA followed by Tukey's test; P < 0.05). [file 7493190.f1.docx]

# Supplementary information

Additional experiments to determine plasma EPA, DHA and free fatty acid (FFA) concentrations were performed in normoglycaemic C57BL/6 mice treated with methyl-GBB for 4 weeks at a dose of 5 mg/kg. Methyl-GBB-treatment resulted in significantly increased EPA and DHA plasma concentrations in fasted (3.4- and 1.9-fold, respectively) and fed (2.5- and 2.3-fold, respectively) mice plasma (Supplementary Fig. 1). Moreover, the concentration of long chain FFAs (sum of C12-C20) was 1.3-fold increased only in fasted, but not fed mouse plasma after treatment with methyl-GBB (Supplementary Fig. 2). Together, selective significant increase in PUFA availability was also present in normoglycaemic mice treated with methyl-GBB, indicating that a pharmacological decrease in fatty acid metabolism is a viable option to increase PUFA availability.

**Supplementary Fig. 1.** The effect of methyl-GBB administration on mouse plasma EPA (A) and DHA (B) concentrations after 4 weeks of treatment at a dose of 5 mg/kg. Each value was calculated as the mean ± S.E.M. of 9 mice. *Significantly different from the respective fed or fasted control group (ANOVA followed by Tukey’s test; P < 0.05).

**Supplementary Fig. 2.** The effect of methyl-GBB administration on mouse plasma FFA concentration (sum of C12-C20 FAs) after 4 weeks of treatment at a dose of 5 mg/kg. Each value was calculated as the mean ± S.E.M. of 9 mice. *Significantly different from the respective fasted control group; # (ANOVA followed by Tukey’s test; P < 0.05).
